# Some of the Present Tendencies in Dentistry

**Published:** 1921-06

**Authors:** C. N. Johnson


### SOME OF THE PRESENT TENDENCIES IN DENTISTRY

BY C. N. JOHNSON, M.A., L.D.S., D.D.S.

Some of the tendencies in operation today are of a very healthy nature. It is a wonderful age in which we live—professionally, industrially and sociologically.

One of the healthy signs of the times in dentistry is the active interest being taken in dental society work. Never in the history of the profession was the attendance at dental meetings so large, nor the interest so keen. Never was there more energy being expended by teachers in our dental colleges than there is today. Never was the demand greater for graduate courses in the various so-called practical branches of our profession, to say nothing of the aggregate of scientific study. All of these tendencies are most encouraging, and they augur well for the future advancement of dentistry.

But there is another phase of the picture—a slight shadow here and there which must needs be somewhat vigorously retouched if we are to come into the full fruition of our aims and ambitions.

In common with the general disorganization of human affairs the world over, there has crept into dentistry certain tendencies which must be met with a bold front and grappled with a strong hand, if we are to prevent a widespread distrust and a grave reflection upon the reputation of our beloved calling. There are not wanting today ample indications that the intelligent public are beginning to raise the question-mark upon some of the prevalent practices of the hour.

One of the tendencies alluded to, while not in itself actively pernicious, is capable of doing great injury unless it is curbed. This relates to the inclination—altogether too widespread—to run to extremes in methods of practice. The profession has suffered more or less from extremists since the earliest days of its history, and why it has not learned a useful lesson from this is one of those inscrutable things incident to human nature. Space does not permit of a detailed consideration of all the various extremes to which the profession has run in the recent past, but a brief mention may be made of one as illustrative of what is happening in several lines of practice.

The great demand for the replacement of partial sets of teeth where a few of the natural organs have been lost, leaving spaces in the arch to be filled by artificial substitutes, has awakened the ingenuity of men to a very wonderful degree. It has resulted in many methods or systems of replacement, each of which has its virtues and its indications in practice. Each commands our respect for its ingeniousness and its applicability to certain cases. The men who have worked out these various systems are entitled to our consideration and commendation—up to a certain point. That point is reached when they become narrow enough to claim universal applicability for their own method to the exclusion of all other methods. The moment a man, however brilliant he may be, sees only one thing in practice, or only one method, that moment he becomes dangerous. And the moment he systematically begins to discredit or tear down other methods to advance the claims of his own that moment he becomes despicable. When will humanity learn that no man ever succeeded in elevating himself by the process of undermining others?

A case in point is worth mentioning. Some of the advocates of the more recent systems of replacement have gone out of their way to attack most strenuously the merits of fixed bridgework. Unquestionably much fixed bridgework in the past has been ill-advised and contraindicated. It has been abused as has been every other method of practice. But to make the sweeping statement or assertion that fixed bridgework is wholly wrong in principle and is always contraindicated, is to go counter to the experience of observant men from the day it was first employed till now. It has been so acceptable to the people, and under its proper indications so serviceable, that to discountenance it entirely in furtherance of the claims of other methods which, no matter what their virtues, have not had anything like the extended trial that has had fixed bridgework, is to exhibit a mental bias which can not be said to be wholly without prejudice. It is safe to assert that had fixed bridgework never been devised there would be countless numbers of those people who for years have enjoyed the blessings of adequate mastication, but who would otherwise have been seriously handicapped in the performance of this importait function. When one considers the lack of judgment in its use the marvel is that it has made as good a record as it has. The real test of any method is its actual service in the mouth over a period of years, and judged by this standard, fixed bridgework has produced ample evidence of its value, and fully justified its existence. One case in point, among many others which might be cited: There died in Chicago, a few weeks ago, a woman who carried to her grave a fixed bridge on her upper teeth which extended from the molar on the one side around the arch to the second bicuspid on the other, with at most five abutments. She had worn this bridge with great comfort for more than a quarter of a century. She was a frail woman when the bridge was made, and her physician had urged that she be provided with some means of adequate mastication. One case does not prove everything, but it is at least safe to assume that if the entire principle of fixed bridgework were wrong this bridge would not have had the record it did. Personally, I am convinced that it prolonged the patient's life.

Why is it that men can not be broadminded? Why do they summarily discard the old and tried, the moment something new and untried has been introduced? We welcome new inventions—we have need of them. But in welcoming the new it is not seemly nor sensible that we aim to sweep away with a wave of the hand all the methods that have gone before. There are indications for the employment of every known method which has in any way proved its merits in practice, and the best interests of dentistry will not be served by a narrow prejudice which sees only one method or one system.

Another tendency of the present I view with even graver apprehension than the one just alluded to. It is an evil which in certain localities has assumed proportions which threaten to react on the profession in a very unfortunate way. I refer to the practice which some men are pursuing, under the guise of modern methods, of exploiting the people and playing on their fears in an altogether unwarranted manner. To explain just what I mean, it will be necessary for me to recite some specific cases in practice. These cases are authentic and I would hesitate to refer to them were it not for the purpose of pointing a moral, and entering a protest. Manifestly there must be a motive behind every professional procedure, and in some of these cases I am not questioning the motive. In others I decidedly am. Since the subject of focal infection has been so much to the fore both in medicine and dentistry, some very unique theories have been propounded and some rather rare methods of practice have been introduced. Many men are perfectly conscientious in the attitude they are assuming and really believe they have just cause for their procedures. They are convinced beyond peradventure that focal infection is at the root of most of the evils connected with human ailments, and they are equally positive that the teeth are the chief culprits in introducing infection into the system. To say to them that the real significance of focal infection in the incidence of disease is not yet definitely settled, or to intimate that undue emphasis has been placed upon the teeth as causative factors in infection is to elicit from them a cry of holy horror, and the charge that the wheels of progress are being blocked. They are convinced in their own minds that they are right, and it is not my purpose to enter into a debate on this very debatable subject. I will merely say parenthetically that it is my firm conviction that while focal infection is a very real and very important factor which can not be ignored in the treatment of disease, and that while the teeth under certain conditions are perfectly capable of spreading infection, yet the role played by infection in causing many of the diseases which are today being attributed to it, and by the teeth as the chief means of bringing about this infection, has been exaggerated out of all proportion to the actual facts in the case. This is something which must be settled in the future on the basis of a longer clinical experience, and a more unbiased observation, and I freely pledge myself that if in the ultimate my position is proved untenable, I will as frankly admit it as I am today questioning some of the prevalent ideas of the hour.

But my chief basis of contention at this time is with the man who, irrespective of his convictions in the matter, is seizing upon the present agitation of this subject to induce people to submit to certain kinds of service which in their saner moods they would never think of having performed. To unduly frighten people about disease of any kind is a grievous wrong, and when people who are in good health are induced to have serviceable teeth extracted on the theory that they may some day cause trouble, it is carrying so-called precaution to the point of folly. I have known people to be so frightened at the prospect held up to them by dentists that they have had this very thing done, only to regret it later.

The discussion of this question of focal infection and the extraction of teeth can not well be approached without a consideration of the X-ray. So much has already been said upon it that it would seem superfluous to refer to it at this time. And yet the last word has not been said about the X-ray, and its relation to the subject under discussion. The X-ray came to us in a dual capacity. It was at the same time the greatest benefactor and the biggest curse that has ever entered the dental profession. What would we do today without this splendid accessory to our work? We would be really lost without it; and yet it is safe to assert that many of our patients would be infinitely better off today if it had never been invented. Why this apparent discrepancy? It is all due to our faulty interpretation of the X-ray, and to our assumption that it will do more than it actually does. This subject is worthy of more elaboration than can be attempted at this time, but reference must be made to a few pertinent facts which have a decided bearing on the question under consideration. In the first place, the X-ray will not show pus formation, it will not demonstrate alveolar abscess, and it will not prove that infection is present. The only thing the X-ray will do with pulpless teeth is to show varying degrees of density in the bone surrounding them. And yet radiographers everywhere are writing with the most utter abandon such terms upon their X-ray films as ''pus pocket,'' ''abscess,'' ''infection,'' etc. And they are thereby frightening people in a wholly reprehensible manner. The mental strabismus that the introduction of the X-ray has caused to many practitioners constitutes itself one of the great evils of the day, and until men can find their balance and gain a proper perspective in their interpretation of the X-ray it will continue to do untold harm.

But worse than all this is the manifest tendency among certain members of our profession to exploit the people through the medium of the X-ray. They take advantage of the ignorance of the public on this subject, and make diagnoses which they know in their hearts are not correct. They write ''abscess'' at the roots of teeth with living pulps, and they write ''pyorrhea'' or ''alveolar absorption'' when they can not write anything else. All of this is not due to misconception on their part—some of it is due to chicanery. As one eminent radiographer has remarked: ''It is a very brave X-ray man who will write on a picture 'I find nothing pathological.' ''

To place such formidable terms as ''abscess,'' ''infection,'' etc., on a radiograph and hand it to a patient is little short of criminal unless there is a moral certainty that they are correct, but this is being done daily in the most off-hand and routine manner. When will members of the profession awaken to their grave responsibility in this important matter and be more conservative in their treatment of their patients?

I promised to recite a few cases in practice to illustrate an unfortunate tendency toward playing upon the credulity of the public. They are selected at random from among very many which might be cited. A lady patient who was not feeling well was referred by her physician to a man who was making a specialty of radiography and oral surgery. His diagnosis was that an upper first permanent molar should be extracted because it was abscessed; that an upper third molar should be removed because it had three cavities and was not worth filling; and that there was a serious alveolar absorption between several of the other teeth. The facts were these: The first permanent molar had a living pulp; the third molar had not a cavity at all, but carried a large filling which was in good condition; and the alveolar absorption was not in the slightest degree more pronounced than one would ordinarily find in a woman of her age. In fact the mouth was an unusually healthy one and showed not the slightest trace of pathology. And yet that man had given this diagnosis to a woman who was so susceptible to impressions that to look her in the face and tell her that she appeared very ill would be to result in her illness in a few minutes. Is that right? Is it professional?

Another was a case of a gentleman who was fortunately not impressionable. He had been ill, and was sent to an X-ray man for radiographs of his teeth. These were submitted to a practitioner, who condemned the teeth—conscientiously condemned them. The patient was finally sent to me and I did not agree with the findings of the other practitioner. In this conflict of opinion the patient naturally sought the advice of various men. One day he wrote me: "They are making a bacteriological examination of the contents of my mouth to see if they find the streptococcus. If they find it I will let you know." I wrote back: "My dear sir, I predict that they will find the streptococcus for the simple reason that it may be found at times in every mouth, healthy or otherwise."

The point I make is this—that the man who knew enough to make a bacteriological examination at all, knew very well that he would find the streptococcus. From the bottom of my heart I hate this kind of posing on the part of professional men, and I propose to fight it as long as I am able to fight anything. Incidentally the man recovered from his illness, and equally incidentally he has all of his condemned teeth.

Speaking of posing reminds me of one case which, were it not for the danger it involves of reflecting on the profession, is sufficiently farcical to elicit nothing but a laugh. A lady developed a toothache, and in the absence of her regular dentist she applied to another for relief. The dentist so consulted was very impressive in his demeanor, and told her that before making an application for the relief of pain in her tooth it would be necessary to make a careful examination of her heart. The lady has been trained to accept implicitly the judgment of her professional adviser, and submitted to the examination, after which the application was made to the tooth. Contrary to all the accepted laws of scientific treatment the tooth still continued to ache, and the patient was obliged to apply to the dentist the following day for relief. Again she was informed that the heart examination would be necessary, and again this important function was performed. When the tooth still persisted in paining after all this marvelous display of professional skill, the patient concluded that it was beyond the reach of human agency and refrained from further consultation. Comment on this case is unnecessary, except to suggest that occurrences such as this can not be considered conducive to respect for the profession on the part of the public.

One other tendency in the profession for which we may exhibit greater charity is the one already alluded to wherein conscientious men are going to extremes which can not be considered safe and sane. The agitation about pulpless teeth and focal infection has thrown many worthy men off their balance and driven them to conclusions which are unfortunate. As has already been intimated, this question has not yet been definitely settled, and all right thinking men should remain open to conviction, but in the light of our clinical experience from the time pulpless teeth began to be filled till now, and with the record that myriads of these teeth have made for efficient and satisfactory service, to contend, as is sagely being done in certain quarters today, that all pulpless teeth should be extracted is to carry radicalism beyond the bounds of reason.

Likewise to advocate the curettage of all sockets from which pulpless teeth have been removed, the general practice of taking stitches in the gum where teeth have been extracted without serious laceration—in other words, the so-called "surgical removal of teeth," must have a better basis of argument than that being put forth by its advocates today.

The prevalent procedure of opening into the gum and bony tissues where the teeth have been out for years in the search for infective areas which are supposed to cause rheumatism must be classed under the same category of extravagant practice. While we may respect the personality of the men who do these things, we can not have the highest regard for their judgment.

To prove that I am drawing no fanciful picture of what is actually being done, permit me to refer to two incidents which have recently come under my notice. Both came from conscientious men, men who are entitled to the respect of their fellow-man so far as their intentions are concerned. One claimed in public that he had opened into the bony tissues of an edentulous mouth for the relief of rheumatism in a case where the teeth had been extracted fifteen years previously. The other remarked that he intended to extract a tooth from the mouth of a member of his own family in which he had some time ago placed an inlay. He recalled that the cavity was large and came near the pulp, and on the possibility that this pulp might some day die and the tooth thus become pulpless he proposed to eliminate it from the mouth.

Gentlemen, I have the prediction to make that our present age will some day be referred to as one of the greatest idiocy in the history of the profession; and yet in the same sentence I would like to revert to an intimation made in the early part of the paper, wherein the inference was suggested that some of our present day tendencies were encouraging in the extreme, and that the prospect for the future was one to which we might look with the utmost confidence. I have the conviction that the ultimate good sense of my profession will carry it safely over the few aberrations which today tend to obscure its horizon, and that we will go safely and surely on to even greater attainments than we have ever achieved in the past.

The present era marks the dawning of a better day in dentistry, when the members of the profession will acquire a broader vision, and achieve a higher concept of their obligations as professional men in their relationship to society at large; when our efforts will be directed more and more towards checking disease at its source; when prevention shall be the watchword of the hour and the terrible conflagration of decay and disease shall be wiped from the mouths of the people. Till this day comes let us resolutely put our shoulders to the wheel, determined to do the utmost in our power to utilize the knowledge and the means at our present command, in a loyal, unselfish service to the people who are committed to our care. If we do this, even though we may make mistakes, we will at least absolve ourselves from the charge that we have been remiss in our professional obligations.—*Pacific Dental Gazette.*

